# Effective dose to immune cells combined with platelet-to-lymphocyte ratio predicts lymphopenia and prognosis in unresectable locally advanced non-small cell lung cancer

**DOI:** 10.3389/fimmu.2025.1657972

**Published:** 2025-09-24

**Authors:** Haoting Yang, Jiachun Ma, Chen Tian, Shunshun Bao, Jingxin Zhang, Fei Wang, Ying Xu, Jinming Yu, Dawei Chen

**Affiliations:** ^1^ Department of Radiation Oncology and Shandong Provincial Key Laboratory of Precision Oncology, Shandong Cancer Hospital and Institute, Shandong First Medical University and Shandong Academy of Medical Sciences, Jinan, Shandong, China; ^2^ Shandong Provincial Key Laboratory of Precision Oncology, Shandong Cancer Hospital and Institute, Shandong First Medical University and Shandong Academy of Medical Sciences, Jinan, Shandong, China; ^3^ Department of Radiation Oncology, Shandong Cancer Hospital and Institute, Shandong First Medical University and Shandong Academy of Medical Sciences, Jinan, Shandong, China

**Keywords:** effective dose to immune cells, platelet-to-lymphocyte ratio, non-small cell lung cancer, chemoradiotherapy, immunotherapy, radiation-induced lymphopenia

## Abstract

**Background:**

In the management of unresectable locally advanced non-small cell lung cancer (LA-NSCLC), the lack of reliable predictive biomarkers for grade ≥ 3 radiation-induced lymphopenia (RIL3+) and prognosis remains a major challenge. This study aims to investigate whether effective dose to immune cells (EDIC) combined with pre-radiotherapy (RT) peripheral blood inflammatory indicators (PBIIs), especially platelet-to-lymphocyte ratio (PLR), could better predict RIL3+ and prognosis in patients with unresectable LA-NSCLC in the immunotherapy era.

**Methods:**

We enrolled 139 patients with unresectable LA-NSCLC who received chemoradiation and consolidation immunotherapy. Logistic regression was used to identify the predictors of RIL3+. Spearman correlation analyses were used to estimate the correlations between each indicator and absolute lymphocyte count (ALC) nadir. Receiver operating characteristic (ROC) curves were used to determine the predictive performance and optimal cut-off of each indicator. Patients were then divided into low- and high-risk groups based on the above cut-offs. Cox proportional hazards regression was used to determine prognostic factors for progression-free survival (PFS) and overall survival (OS). Survival outcomes were assessed using Kaplan–Meier methods.

**Results:**

Logistic regression showed that both EDIC (*P* = 0.002) and PLR (*P* < 0.001) were significantly associated with RIL3+. ROC curves showed the highest predictive power of the PLR among the PBIIs. Spearman correlation analysis showed that both EDIC (*P* < 0.001) and PLR (*P* < 0.001) were significantly correlated with ALC nadir. Compared to the model using EDIC (*P* = 0.026) or PLR (*P* = 0.021) alone, the combination of EDIC and PLR showed superior predictive performance. The optimal cut-offs of EDIC and PLR were 4.44 Gy and 107.70, respectively. The incidence rates of RIL3+ in the low- and high-risk groups were 44.3% and 90.0%, respectively (*P* < 0.001). Compared to the high-risk group, patients in the low-risk group had a longer median PFS (*P* = 0.011) and OS (*P* = 0.013).

**Conclusions:**

In the immunotherapy era, the combination of EDIC and pre-RT PLR is a predictive biomarker of RIL3+ and prognosis in patients with unresectable LA-NSCLC. Reducing EDIC and considering pre-RT PLR may potentially avoid RIL3+ and improve prognosis.

## 1 Introduction

Lung cancer is the leading cause of cancer-related death worldwide, and unresectable locally advanced non-smaldl cell lung cancer (LA-NSCLC) accounts for 25%-30% of all lung cancer (1). The PACIFIC and GEMSTONE 301 trials established consolidation immunotherapy as the standard of care for patients with unresectable LA-NSCLC following concurrent or sequential chemoradiotherapy (2, 3). With the addition of immunotherapy, the 5-year overall survival (OS) of unresectable LA-NSCLC patients has increased from 33.4% to 42.9% (2). However, the radiation-induced lymphopenia (RIL) has emerged as an important issue in the management of unresectable LA-NSCLC, especially in the era of immunotherapy. Previous studies have shown that grade ≥ 3 radiation-induced lymphopenia (RIL3+) has a negative impact on the recurrence and survival in non-small cell lung cancer (NSCLC) patients (4–6). Reliable predictors for RIL3+ are urgently needed in unresectable LA-NSCLC management, as early identification of high-risk patients enables timely interventions to prevent severe lymphopenia and optimize survival outcomes.

It is well known that the excessive radiation dose to the lungs, heart and whole body are related to lymphopenia and poor prognosis of NSCLC patients (7–9). In a secondary analysis of RTOG 0617, Jin et al. (10) proposed a model of radiation dose to the immune system, called the effective dose to immune cells (EDIC). The EDIC was calculated based on the equivalent uniform dose to the entire blood, considering radiation doses to all blood-containing organs, blood flow, and the fractionation effect. EDIC estimates the dose to immune cells by calculating the radiation dose to the circulating blood as a surrogate, with contributions from each blood-containing organ, including the lungs and heart, and large and small blood vessels. Following the development of this model, several studies have shown that an increase in EDIC is associated with a higher incidence of RIL3+ and poorer prognosis in unresectable LA-NSCLC patients (11–14). However, the ability of EDIC alone to predict RIL3+ was limited, with an area under the curve (AUC) of less than 0.6 (14). Further research is needed to determine which indicators could be potential partners for EDIC to improve predictive capabilities.

The peripheral blood inflammatory indicators (PBIIs) are easily accessible and closely associated with the prognosis of lung cancer patients, including neutrophil-to-lymphocyte ratio (NLR), derived neutrophil-to-lymphocyte ratio (dNLR), lymphocyte-to-monocyte ratio (LMR), platelet-to-lymphocyte ratio (PLR), and systemic immune-inflammation index (SII) (15–19). The PLR may reflect the inflammatory state of the body and the balance between pro-tumor and anti-tumor, which is a promising indicator for assessing efficacy and prognosis in lung cancer patients (17, 18). In metastatic NSCLC patients receiving nivolumab, higher pre-treatment PLR was associated with inferior progression-free survival (PFS) and OS (18). The predictive performance of PLR for RIL3+ and prognosis in patients with unresectable LA-NSCLC remains to be explored.

In this study, we investigated whether EDIC combined with pre-radiotherapy (RT) PBIIs, especially PLR, could better predict RIL3+ and prognosis in patients with unresectable LA-NSCLC in the era of immunotherapy.

## 2 Patients and methods

### 2.1 Study population and treatment

A total of 139 patients with unresectable LA-NSCLC who received chemoradiotherapy (CRT) and consolidation immunotherapy between January 2019 and December 2021 were retrospectively enrolled at Shandong Cancer Hospital and Institute. According to the AJCC 8th TNM staging of lung cancer, patients were staged with either PET/CT or CT imaging of the chest, abdomen, and brain MRI. Inclusion criteria were as follows: (1) patients with complete blood count data at baseline (within 1 week before RT) and weekly assessments during RT; (2) the RT fraction number ≥ 25; (3) Eastern Cooperative Oncology Group (ECOG) performance status of 0 to 1; (4) negative for driver genes. Exclusion criteria were: (1) patients with more than one primary tumor; (2) incomplete treatment and follow-up information; (3) early termination of RT before reaching 45 Gy. All patients underwent chemotherapy and intensity-modulated radiation therapy (IMRT), followed by consolidation immunotherapy. Subsequently, follow-up imaging studies were performed every 2–3 months for the first 3 years after CRT and every 6 months from the third year.

The study was approved by the Ethics Committee of Shandong Cancer Hospital and Institute (Approval Number: SDTHEC202410056) and followed the Declaration of Helsinki. Due to the retrospective nature of this study, the committee waived the requirement for informed consent. All patient data were fully anonymized and all direct identifiers were removed to protect patient confidentiality.

### 2.2 Grade of lymphopenia

According to the Common Terminology Criteria for Adverse Events (CTCAE) version 5.0, absolute lymphocyte count (ALC) of >1.00 × 10^9^ cells/L, 0.80-1.00 × 10^9^ cells/L, 0.50-0.80 × 10^9^ cells/L, 0.20-0.50 × 10^9^ cells/L, and < 0.20 × 10^9^ cells/L were defined as grade G0, G1, G2, G3, and G4 lymphopenia, respectively. Severe lymphopenia was defined as grade G3 and G4 lymphopenia.

### 2.3 Calculation of EDIC

Dosimetric data of RT were extracted from Eclipse (Varian Medical Systems). According to the model developed by Ladbury et al. (12), EDIC was calculated as a function of mean lung dose (MLD), mean heart dose (MHD), mean body dose (MBD), and number of RT fractions (n). The formula was as follows:


$$EDIC=0.12\times MLD+0.08\times MHD+[0.45+0.35\times 0.85\times (\sqrt{\frac{n}{45}})]\times MBD$$

### 2.4 Calculation of PBIIs

The NLR, PLR, and LMR were calculated as neutrophil count/lymphocyte count, platelet count/lymphocyte count, lymphocyte count/monocyte count, respectively. The dNLR and SII were calculated using the formulas below:


$$dNLR=\frac{\text{neutrophil count}}{\text{leukocyte count }-\text{ lymphocyte count}}$$

$$SII=\frac{\text{platelet count}\times \text{neutrophil count}}{\text{lymphocyte count}}$$

### 2.5 Statistical analysis

The primary endpoint was RIL3+. The secondary endpoints were PFS and OS. The PFS was defined from the date of RT initiation to the date of tumor progression, death, or last follow-up. The OS was defined from the start of RT until death or last follow-up. The pre-RT PBIIs was calculated using the blood count data within 1 week prior to RT. The correlations of ALC nadir with both EDIC and PLR were evaluated using Spearman correlation analysis. Univariate and multivariate logistic regression analyses were used to identify predictors with RIL3+. Receiver operating characteristic (ROC) curves were plotted to compare the predictive performances of different indicators. We evaluated the area under the curve (AUC) values for each ROC curve internally using 1,000 bootstrap samples obtained from the original dataset by sampling the same individuals multiple times. ROC curves were compared using DeLong’s test. Youden’s index was used to determine the optimal cut-offs of EDIC and PLR. Each indicator was then assigned a point of 0 or 1 based on the cut-off. A point of 0 was given for a value below the cut-off, and a point of 1 for not less than the cut-off. The sum of the points for each indicator yielded a total score of 0, 1, or 2, which divided patients into a low-risk group (total score = 0-1) and a high-risk group (total score = 2). Pearson’s chi-squared test was used to compare categorical variables. Univariate Cox proportional hazards regression analyses were used to determine prognostic factors for PFS and OS. Survival curves were estimated using the Kaplan-Meier method and compared using the log-rank test. All tests were two-sided and *P* < 0.05 was considered statistically significant. Statistical analyses were conducted using SPSS software version 26 (IBM Corporation, NY, USA) and R version 4.3.3 (R Foundation for Statistical Computing, Vienna, Austria).

## 3 Results

### 3.1 Patient characteristics

Baseline characteristics of the 139 patients were listed in **Table 1**. In our cohort, the vast majority of patients were male (n = 117, 84.2%). Most patients were stage III; (n = 127, 91.4%) and a small percentage of patients were stage II (n = 12, 8.6%). The histology of most patients is squamous cell carcinoma (n = 94, 67.6%). A median radiation dose of 60 Gy (interquartile range [IQR], 58–60 Gy) was delivered in 30 fractions, five days a week. The median EDIC was 1.83 Gy (IQR, 1.22 – 2.59 Gy), and the median PLR was 147.22 (IQR, 105.27 – 203.64). The baseline ALC was 1.55 × 10^9^ cells/L (IQR, 1.17 – 1.94 × 10^9^ cells/L). The majority of patients received concurrent chemoradiotherapy (CCRT) (n = 97, 69.8%) while others received sequential chemoradiotherapy (SCRT) (n = 42, 30.2%).

**Table 1:** Patient and treatment characteristics.

Variables	n*
Age, y	68 (61–73)
Gender	
Male	117 (84.2%)
Female	22 (15.8%)
Smoking history	
No	61 (43.9%)
Yes	78 (56.1%)
ECOG performance status	
0	80 (57.6%)
1	59 (42.4%)
BMI	
< 25	88 (63.3%)
≥ 25	51 (36.7%)
T stage	
T1	21 (15.1%)
T2	42 (30.2%)
T3	22 (15.8%)
T4	54 (38.8%)
N stage	
N0	14 (10.1%)
N1	17 (12.2%)
N2	64 (46.0%)
N3	44 (31.7%)
TNM stage	
IIA-IIB	12 (8.6%)
IIIA	48 (34.5%)
IIIB	62 (44.6%)
IIIC	17 (12.2%)
Histology	
Adenocarcinoma	41 (29.5%)
Squamous cell carcinoma	94 (67.6%)
Others	4 (2.9%)
Chemoradiotherapy	
CCRT	97 (69.8%)
SCRT	42 (30.2%)
Chemotherapy regimen	
Pemetrexed + Platinum	39 (28.1%)
Paclitaxel + Platinum	69 (49.6%)
Others	31 (22.3%)
Immunotherapy type	
Anti-PD-1 immune checkpoint	120 (86.3%)
Anti-PD-L1 immune checkpoint	19 (13.7%)
PTV, cc	242.70 (159.25-354.35)
Radiation dose, Gy	60 (58–60)
EDIC, Gy	4.87 (3.56-6.20)
Baseline inflammatory blood markers	
NLR	2.47 (1.58-3.65)
dNLR	1.77 (1.17-2.70)
LMR	3.42 (2.53-5.50)
PLR	147.22 (105.27-203.64)
SII	555.47 (316.80-834.59)

ECOG, Eastern Cooperative Oncology Group; BMI, body mass index; CCRT, concurrent chemoradiotherapy; SCRT, sequential chemoradiotherapy; PD-1, programmed death 1; PD-L1, programmed cell death ligand 1; PTV, planning target volume; ALC, absolute lymphocyte count; EDIC, effective dose to immune cells; NLR, neutrophil-to-lymphocyte ratio; dNLR, derived neutrophil-to-lymphocyte ratio; LMR, lymphocyte-to-monocyte ratio; PLR, platelet-to-lymphocyte ratio; SII, systemic immune-inflammation index.

*Values are number (percentage) or median (IQR).

### 3.2 Factors associated with RIL3+

In our study, 89 (64.0%) patients experienced RIL3+ during RT. Patients with non-RIL3+ had longer median PFS (*P* = 0.014) and OS (*P* = 0.023) compared to patients with RIL3+ (**Supplementary Figures S1A, B**). Then univariate and multivariate logistic regression analyses were performed to determine the factors associated with RIL3+ (**Table 2**). The univariate analysis revealed that gender, histology, EDIC, PLR, and planning target volume (PTV) were associated with RIL3+. After including the above variables in the multivariate analysis, gender (odds ratio [OR] = 7.42; 95% confidence interval [CI], 1.30 – 42.39; *P* = 0.024), EDIC (OR = 1.60; 95% CI, 1.18 – 2.16; *P* = 0.002), and PLR (OR = 1.02; 95% CI, 1.01 – 1.03; *P* < 0.001) were significantly related with the occurrence of RIL3+. People with higher EDIC or PLR were more likely to experience RIL3+. There were no statistically significant differences in EDIC (*P* = 0.179) or PLR (*P* = 0.720) between CCRT and SCRT groups (**Supplementary Figure S2**).

**Table 2:** Univariate and multivariate logistic regression analysis for RIL3+ during radiotherapy.

Variables	Univariate analysis		Multivariate analysis	
	OR (95% CI)	*P*	OR (95% CI)	*P*
Age				
< 65	1.00 (Reference)			
≥ 65	0.99 (0.49-2.02)	0.981		
Gender				
Male	1.00 (Reference)		1.00 (Reference)	
Female	6.96 (1.55-31.15)	0.011	7.42 (1.30-42.39)	0.024
Smoking history				
No	1.00 (Reference)			
Yes	0.60 (0.30-1.23)	0.162		
ECOG performance status				
0	1.00 (Reference)			
1	0.70 (0.35-1.41)	0.322		
BMI				
< 25	1.00 (Reference)			
≥ 25	0.70 (0.34-1.43)	0.331		
TNM stage				
IIA-IIB	1.00 (Reference)			
IIIA	1.18 (0.33-4.19)	0.796		
IIIB	2.44 (0.70-8.60)	0.164		
IIIC	3.25 (0.66-15.98)	0.147		
Histology				
ADC	1.00 (Reference)		1.00 (Reference)	
SCC	0.38 (0.16-0.88)	0.025	0.59 (0.20-1.75)	0.342
Others	0.84 (0.08-9.13)	0.889	1.00 (0.07-14.10)	0.999
Chemotherapy regimen				
Pemetrexed + Platinum	1.00 (Reference)			
Paclitaxel + Platinum	0.50 (0.21-1.20)	0.121		
Others	0.55 (0.20-1.51)	0.245		
EDIC (per 1 Gy)	1.46 (1.17-1.83)	< 0.001	1.60 (1.18-2.16)	0.002
Pre-RT NLR	1.21 (0.99-1.48)	0.065		
Pre-RT dNLR	1.34 (1.00-1.81)	0.053		
Pre-RT LMR	1.00 (0.96-1.04)	0.979		
Pre-RT PLR	1.01 (1.01-1.02)	0.002	1.02 (1.01-1.03)	< 0.001
Pre-RT SII	1.00 (1.00-1.00)	0.135		
PTV (cc)	1.01 (1.01-1.01)	0.010	1.00 (1.00-1.01)	0.100

ECOG, Eastern Cooperative Oncology Group; BMI, body mass index; ADC, adenocarcinoma; SCC, squamous cell carcinoma; EDIC, effective dose to immune cells; RT, radiotherapy; NLR, neutrophil-to-lymphocyte ratio; dNLR, derived neutrophil-to-lymphocyte ratio; LMR, lymphocyte-to-monocyte ratio; PLR, platelet-to-lymphocyte ratio; SII, systemic immune-inflammation index; PTV, planning target volume; OR, odds ratio; CI, confidence interval.

**Figure 1:**
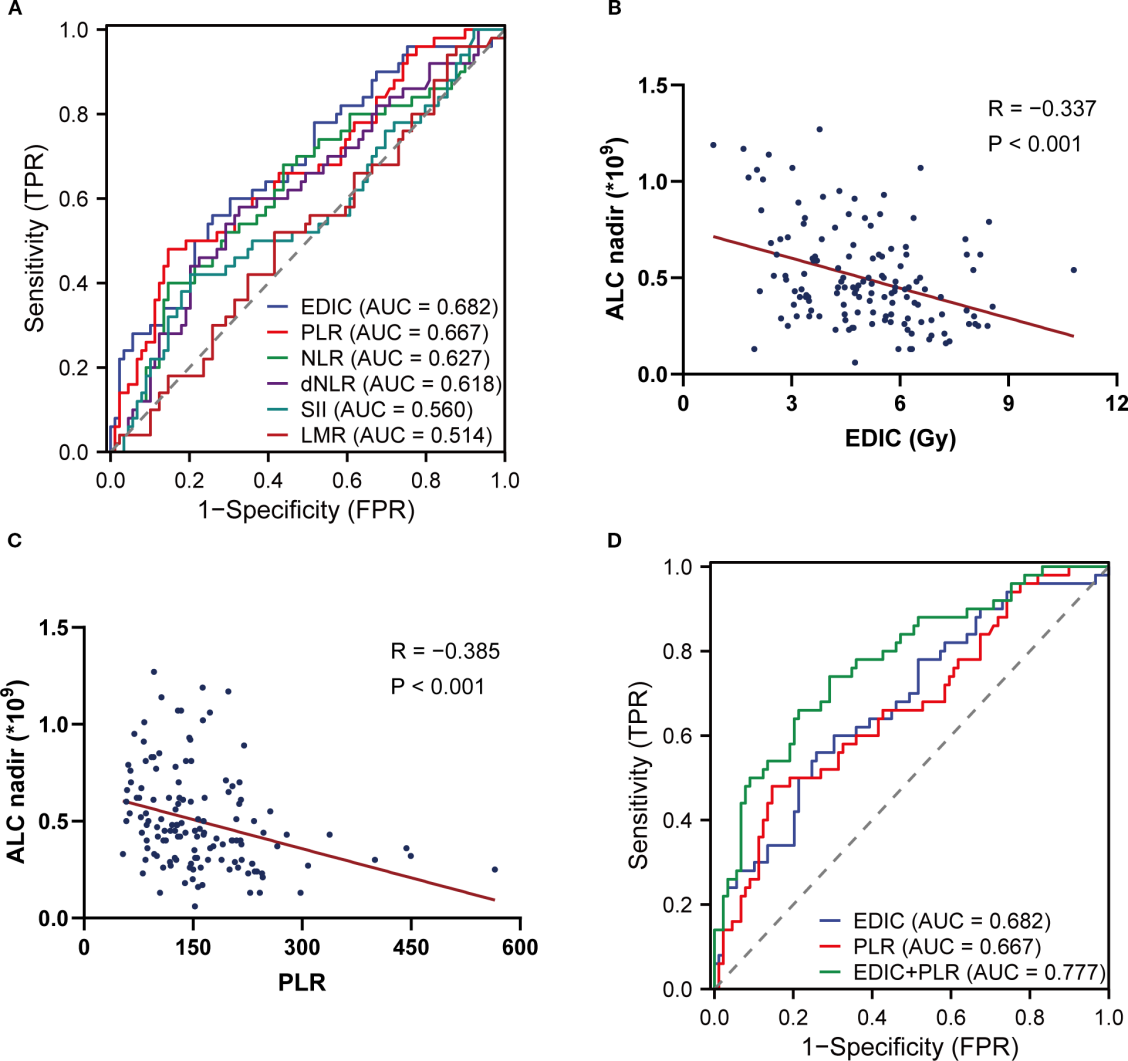
The combination of EDIC and pre-RT PLR predicts RIL3+. The ROC curves of EDIC and PBIIs **(A)**. Spearman correlation analyses between EDIC **(B)**, PLR **(C)** and the ALC nadir during RT. The comparison of ROC curves of EDIC, PLR and their combination **(D)**. EDIC, effective dose to immune cells; RT, radiotherapy; PLR, platelet-to-lymphocyte ratio; RIL3+, grade ≥ 3 radiation-induced lymphopenia; ROC, receiver operating characteristic; PBIIs, peripheral blood inflammatory indicators; ALC, absolute lymphocyte count.

The ROC curves showed that EDIC (AUC = 0.682) had the highest predictive value for predicting RIL3+ during RT (**Figure 1A**). Among PBIIs, PLR (AUC = 0.667) had the highest predictive value for RIL3+. Spearman correlation analysis revealed that both EDIC (R = -0.337, *P* < 0.001) and PLR (R = -0.385, *P* < 0.001) were associated with ALC nadir (**Figures 1B, C**). Compared to individual models using EDIC (AUC = 0.682; *P* = 0.026) or PLR (AUC = 0.667; *P* = 0.021) alone, the combination of EDIC and PLR demonstrated superior accuracy (AUC = 0.777) in predicting RIL3+ (**Figure 1D**). The optimal cut-offs of EDIC and PLR were 4.44 Gy and 107.70, respectively. The optimism-corrected bootstrap AUC values were in **Supplementary Table S1**.

### 3.3 Risk groups based on EDIC and pre-RT PLR predicted RIL3+ and prognosis

**Figure 2:**
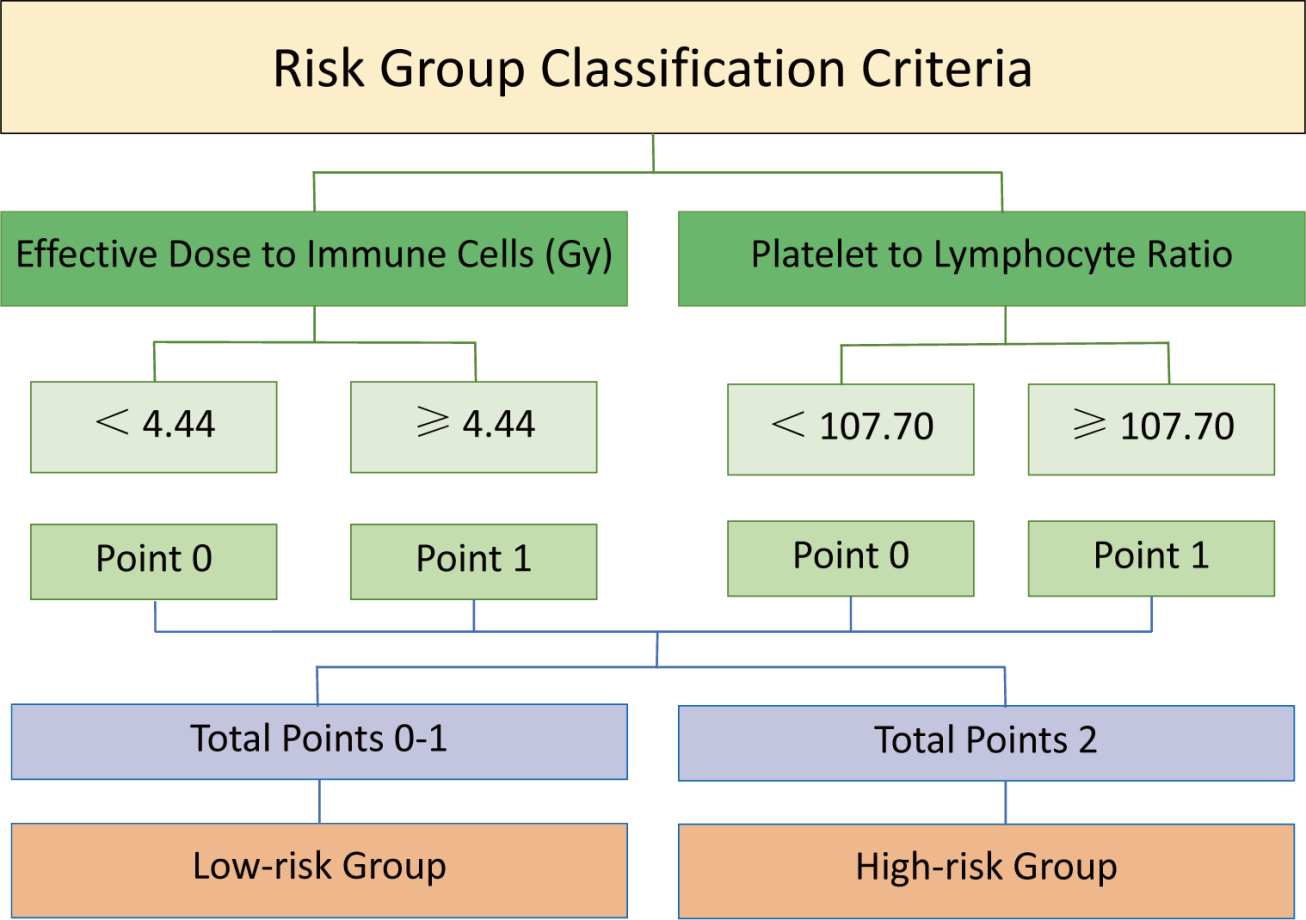
Schematic diagram of risk group division based on EDIC and PLR. Each indicator was assigned a point of 0 or 1 based on the cut-off. A point of 0 was given for a value below the cut-off, and a point of 1 for not less than the cut-off. The sum of the points for each indicator yielded a total score of 0, 1, or 2, which divided patients into a low-risk group (total score = 0-1) and a high-risk group (total score = 2). EDIC, effective dose to immune cells; PLR, platelet-to-lymphocyte ratio.

The risk groups were divided based on the optimal cut-offs of EDIC and PLR (**Figure 2**). A detailed description of the specific grouping method can be found in **Supplementary Figure S3**. Each indicator was assigned a point of 0 or 1 based on the cut-off. For instance, patients with EDIC values < 4.44 or PLR values < 107.70 were scored as 0, while EDIC values ≥ 4.44 or PLR values ≥ 107.70 were scored as 1. Specifically, the high-risk group was defined as patients with EDIC ≥ 4.44 Gy and PLR ≥ 107.70, while the remaining patients were in the low-risk group. A comparison of baseline information between low-risk and high-risk groups was shown in **Supplementary Table S2**. We found no statistically significant differences in immunotherapy cycles (*P* = 0.064) or chemotherapy cycles (*P* = 0.119) between the high-risk and low-risk groups.

**Figure 3:**
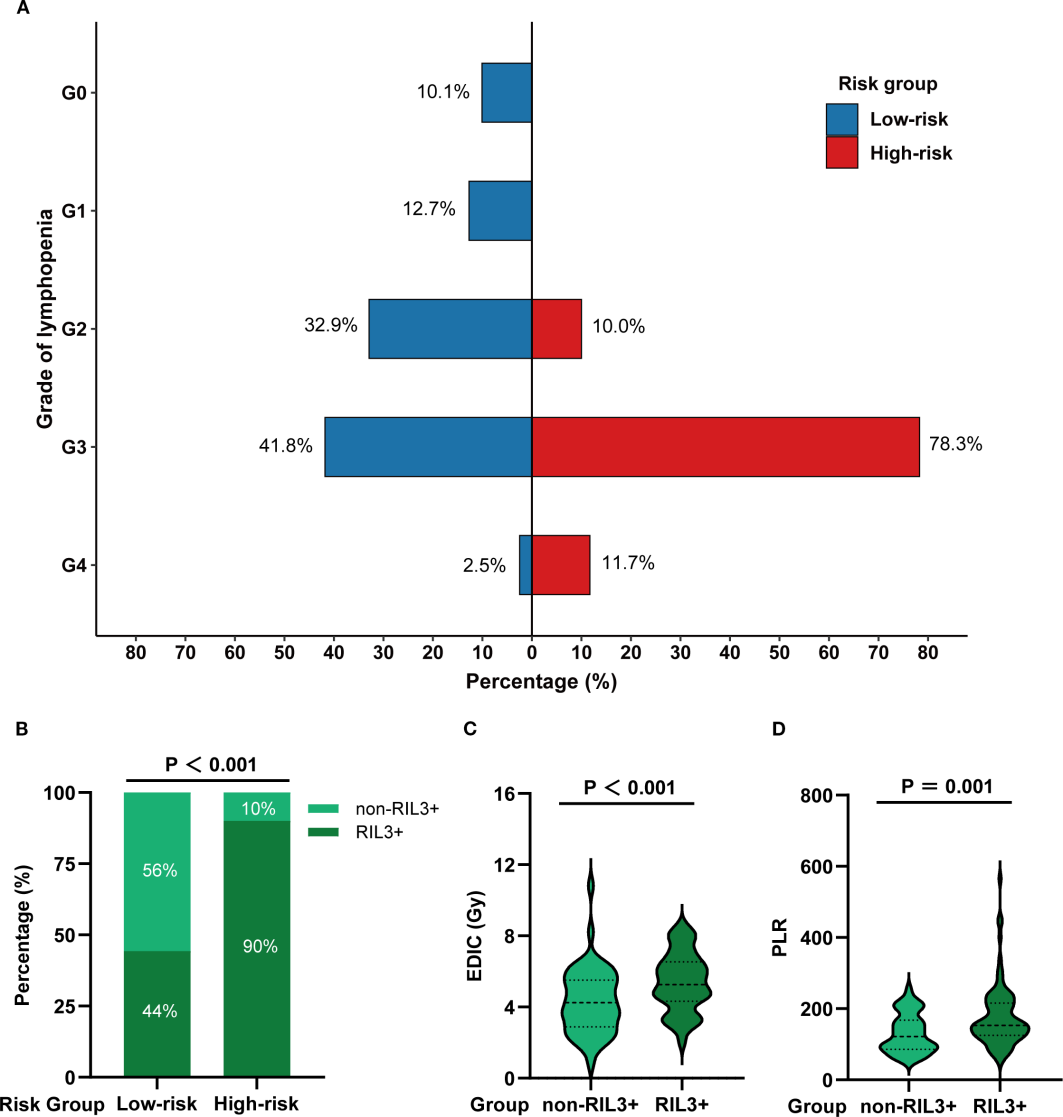
A comparison of the incidence of RIL3+ in different risk groups. The grade distribution of RIL in different risk groups **(A)**. The incidence of non-RIL3+ and RIL3+ in different risk groups **(B)**. Comparison of EDIC **(C)** and PLR **(D)** in non-RIL3+ and RIL3+ groups. RIL3+, grade ≥ 3 radiation-induced lymphopenia; RIL, radiation-induced lymphopenia; EDIC, effective dose to immune cells; PLR, platelet-to-lymphocyte ratio.

**Figure 4:**
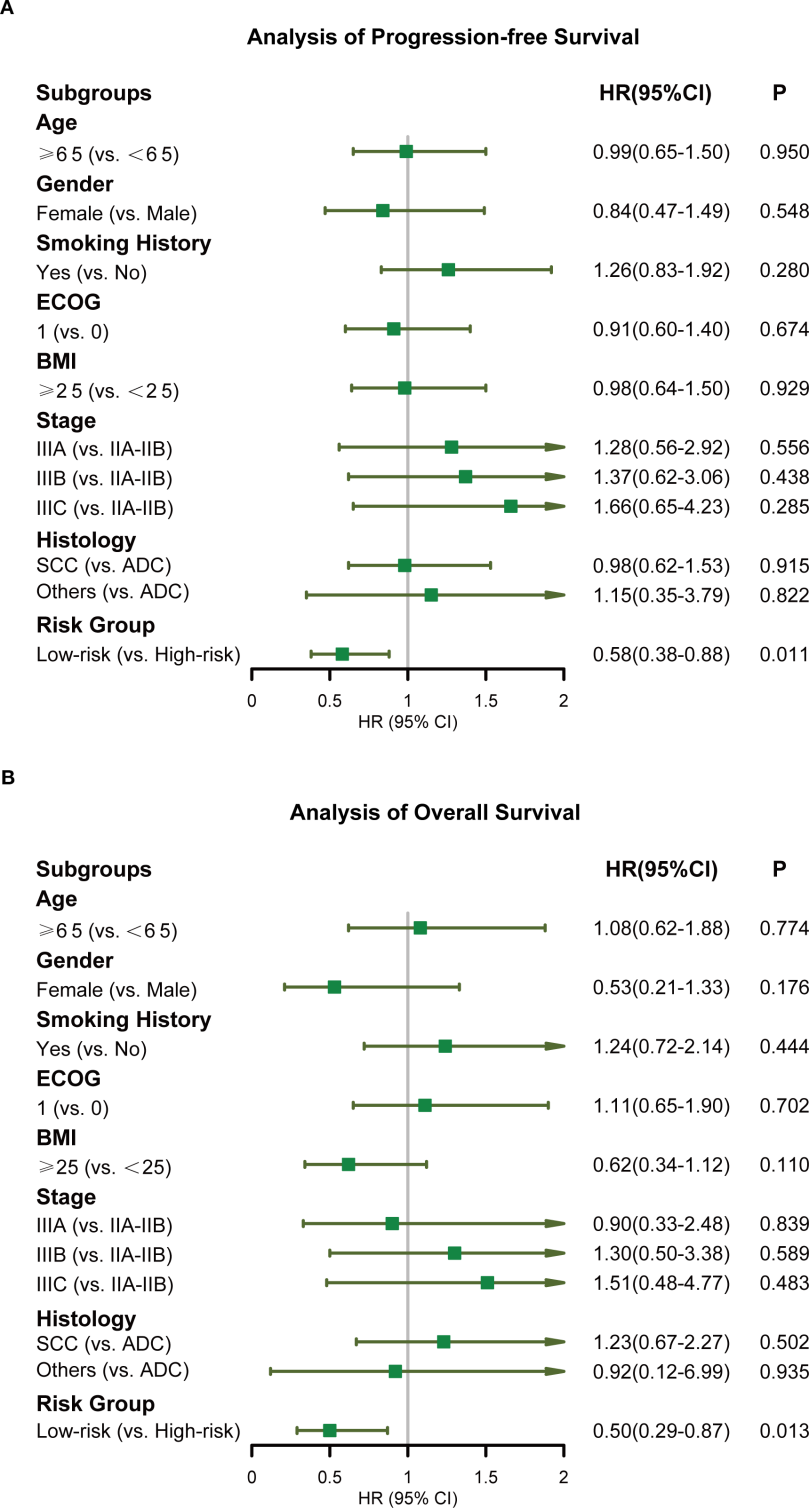
Forest plots displaying the Cox regression analysis for PFS and OS. PFS **(A)**, OS **(B)**. PFS, progression-free survival; OS, overall survival.

**Figure 5:**
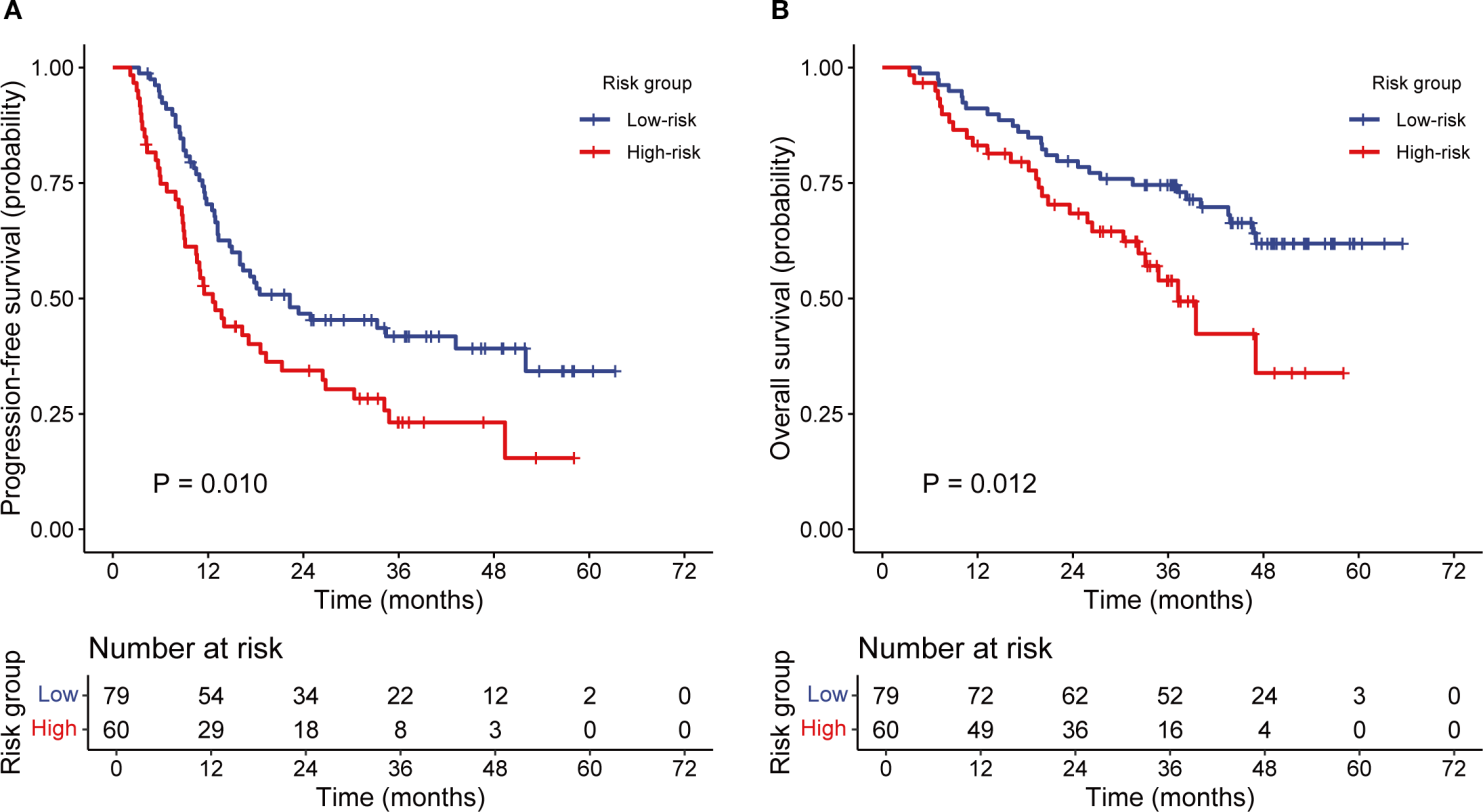
The Kaplan–Meier analysis of PFS and OS according to the risk groups. PFS **(A)**, OS **(B)**. PFS, progression-free survival; OS, overall survival.

The incidence rates of RIL3+ in the low- and high-risk groups were 44.3% and 90.0%, respectively (*P* < 0.001; **Figures 3A, B**). Moreover, patients with RIL3+ tended to have higher EDIC (*P* < 0.001, **Figure 3C**) or PLR (*P* = 0.001, **Figure 3D**) than those with non-RIL3+. The median PFS of our cohort was 16.4 months (95% CI, 13.2 – 24.9 months), while median OS was not reached (NR) after a median follow-up of 42.9 months (95% CI, 40.6 – 45.3 months). Univariate Cox proportional hazards regression analyses showed that the risk group was significantly associated with both PFS (*P* = 0.011; **Figure 4A**) and OS (*P* = 0.013; **Figure 4B**). Compared to the high-risk group, the low-risk group had a significantly better PFS (hazard ratio [HR] = 0.58; 95% CI, 0.38 – 0.88; *P* = 0.011) and OS (HR = 0.50; 95% CI, 0.29 – 0.87; *P* = 0.013). The median PFS of the low- and high-risk groups were 22.30 months (95% CI, 15.00 months – NR) and 12.60 months (95% CI, 9.10 – 21.30 months), respectively (*P* = 0.010; **Figure 5A**). The median OS was NR in the low-risk group, whereas the high-risk group exhibited a significantly shorter median OS of 37.25 months (95% CI, 32.27 months – NR) (*P* = 0.012; **Figure 5B**).

### 3.4 Subgroup analysis of patients in the low-risk group

**Figure 6:**
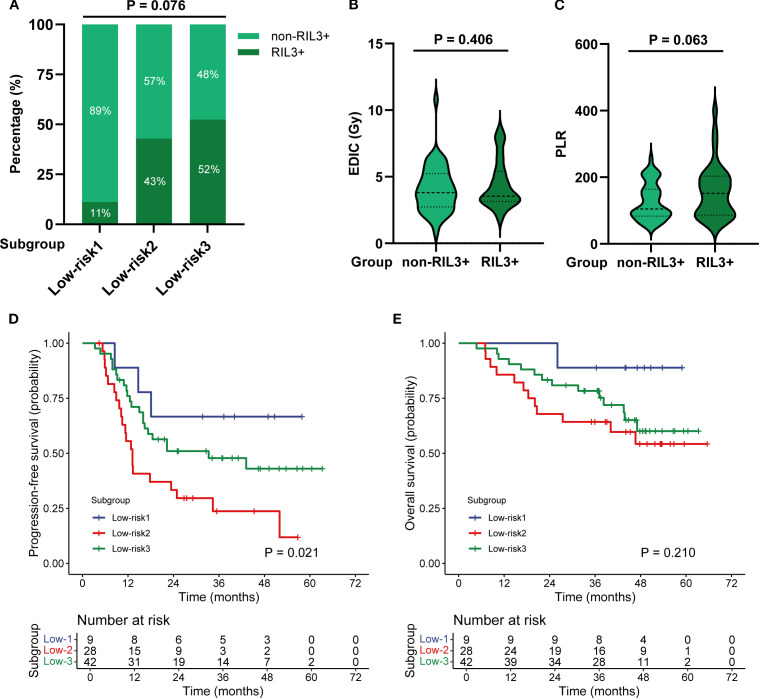
Subgroup analysis of patients in the low-risk group. The incidence of non-RIL3+ and RIL3+ in different subgroups **(A)**. Comparison of EDIC **(B)** and PLR **(C)** in non-RIL3+ and RIL3+ groups. The Kaplan–Meier analysis of PFS **(D)** and OS **(E)** by subgroups. RIL3+, grade ≥ 3 radiation-induced lymphopenia; EDIC, effective dose to immune cells; PLR, platelet-to-lymphocyte ratio; PFS, progression-free survival; OS, overall survival.

The patients in the low-risk group included three subgroups: 1) Low-risk 1: EDIC < 4.44 Gy and PLR < 107.70; 2) Low-risk 2: EDIC ≥ 4.44 Gy and PLR < 107.70; 3) Low-risk 3: EDIC < 4.44 Gy and PLR ≥ 107.70. Subgroup analyses showed that the incidence rates of RIL3+ in the Low-risk 1, Low-risk 2, and Low-risk 3 subgroups were 11.1%, 42.9%, and 52.4%, respectively (*P* = 0.076; **Figure 6A**). EDIC (*P* = 0.406) and PLR (*P* = 0.063) did not significantly differ between RIL3+ and non-RIL3+ patients in the low-risk group (**Figures 6B, C**). The median PFS of the Low-risk 1, Low-risk 2, and Low-risk 3 groups were NR (95% CI, 18.10 months – NR), 13.20 months (95% CI, 10.50 – 34.40 months), and 33.30 months (95% CI, 16.40 months – NR), respectively (*P* = 0.021; **Figure 6D**). The median OS for all three subgroups was NR (*P* = 0.210; **Figure 6E**).

### 3.5 Association between risk groups and toxicity

In our cohort, there were 64 instances of immune-related adverse events (irAEs). Of these, 34 occurred in the low-risk group and 30 in the high-risk group. There was no statistically significant difference in the incidence of irAEs between the two groups (*P* = 0.415, **Supplementary Table S3**). The overall incidence of grade ≥ 2 pneumonitis was 38.1%. The incidence of grade ≥ 2 pneumonitis was significantly higher in the high-risk group (48.3%) compared to the low-risk group (30.4%) (*P* = 0.031, **Supplementary Figure S4**).

## 4 Discussion

Our study is the first to underscore that the combination of EDIC and pre-RT PLR should be considered as a modifiable factor to prevent RIL3+ and improve prognosis. This suggests that efforts to reduce EDIC and consider pre-RT PLR should be emphasized in the management of unresectable LA-NSCLC. In real-world clinical practice, some patients with unresectable LA-NSCLC receive SCRT instead of CCRT due to concerns about tolerability of CCRT, advanced age or frailty, and comorbidities. In contrast to the previously published study by Yang et al. on lymphopenia in patients receiving CCRT, we expanded the study population from those treated with CCRT to those treated with CCRT or SCRT (14). This makes our study more generalizable to clinical practice.

Subgroup analyses of patients in the low-risk group showed further risk stratification for PFS but no statistically significant difference for OS and RIL3+. An interesting trend was observed within the low-risk group: patients with high EDIC but low PLR (low-risk 2 subgroup) had poorer outcomes than those with low EDIC but high PLR (low-risk 3 subgroup). A high EDIC likely causes a profound and sustained suppression of the immune system’s ability to mount an anti-tumor response, which may outweigh the negative prognostic impact of a high PLR (a marker of a pro-tumor inflammatory state) in the short to medium term. This suggests that treatment-induced lymphopenia may be a more dominant negative prognostic factor than baseline systemic inflammation in this setting. However, this finding requires further validation due to the limited sample size in our cohort.

The combination of RT and immunotherapy may have complex interactive effects, with RT both activating and suppressing immunity (20). On the one hand, RT induces immunogenic cell death and promotes T cell-mediated anti-tumor immune responses, producing an *in situ* vaccine effect (21–23); on the other hand, due to the high radiosensitivity of bone marrow hematopoietic stem cells, patients may experience severe bone marrow suppression during RT, resulting in a significant decrease in lymphocyte counts (i.e., lymphopenia) and a corresponding decrease in anti-tumor immune functions (24–26).

A promising approach to reduce the incidence of RIL3+ may be to reduce the number of RT fractions. Patients who have a high chance of RIL3+ during conventional RT may be good candidates for hypofractionated radiotherapy (HFRT). HFRT shortens the course of treatment, resulting in a lower cumulative dose of radiation to normal tissues (including lymphocytes), which helps protect the immune system and improve survival outcomes (27, 28). HFRT also activates the immune response by remodeling the tumor microenvironment and reduce the infiltration of myeloid-derived suppressor cells (MDSCs) (29). In contrast, conventional RT typically induces significant immunosuppression (26).

The use of advanced RT techniques can also reduce the incidence of RIL3+. A previous study reported that patients treated with proton therapy had lower EDIC than those treated with photon therapy (13). Compared to conventional photon therapy, proton therapy demonstrates the potential to reduce the incidence of RIL3+ through its dosimetric advantage in sparing critical organs-at-risk (OARs), including the thoracic vertebrae, major blood vessels, heart, and lungs (30, 31). FLASH-RT, an ultra-high dose rate irradiation, significantly reduces lymphopenia by decreasing the exposure time to circulating blood volume (32, 33). In the near future, advanced RT techniques may serve as ideal partners for combined RT and immunotherapy. This should be considered in patients at high risk of developing RIL3+ during photon therapy.

Our study has several limitations. First, the EDIC model does not include other immune substructures such as bone marrow, spleen, liver, lymph nodes, and lymphatic ducts. These substructures have been shown to contribute to lymphopenia (34). Further efforts should be made to improve the EDIC model by incorporating the radiation dose to the above structures into the formula. Second, unbalanced lymphocyte subtypes may have different effects on prognosis (35). Due to the heterogeneity of radiosensitivity in lymphocyte subpopulations, RT may selectively reduce the number of CD4^+^ T cells and B cells, while having less effect on CD8^+^ T cells (36, 37). Therefore, models based on lymphocyte subtypes rather than total ALC should be established in the future. Third, this was a retrospective study with a small sample size and inevitable heterogeneity of the patients included. The single-center design and ethnically homogeneous cohort may limit the generalizability of our findings, as genetic background, lifestyle, and regional healthcare systems can influence baseline inflammatory marker levels and treatment outcomes. Previous studies on PLR indicate that Europeans generally exhibit higher PLR values than Asians (18, 38–41). Therefore, the prognostic cut-off values for PLR we identified may require validation in more diverse populations. Finally, this study lacks an external validation cohort. To address this limitation, we are planning a prospective multi-center collaboration to validate these results externally. Confirming our findings in these independent cohorts will be a critical step towards the potential clinical application of this prognostic model.

Despite these limitations, our study highlights the combination of EDIC and pre-RT PLR as a potential biomarker for predicting RIL3+ and prognosis in unresectable LA-NSCLC patients receiving CRT and consolidation immunotherapy. Risk stratification based on EDIC and pre-RT PLR is simple, accessible, and cost-effective. This makes it a promising biomarker to assess RIL3+ and prognosis in clinical practice. With further validation and exploration, EDIC and pre-RT PLR could make a significant contribution to individualized therapy by helping to select patients most likely to benefit from consolidation immunotherapy.

## 5 Conclusion

Our study showed that pre-RT PLR had the highest predictive accuracy for RIL3+ among the PBIIs. EDIC and pre-RT PLR were significantly associated with RIL3+ in patients with unresectable LA-NSCLC. Notably, the combination of EDIC and pre-RT PLR better predicted RIL3+ than using either parameter alone. Risk groups based on EDIC and pre-RT PLR were able to predict both PFS and OS. Therefore, reducing EDIC and considering pre-RT PLR may potentially avoid RIL3+ and improve prognosis in the immunotherapy era.

## Data Availability

The raw data supporting the conclusions of this article will be made available by the authors, without undue reservation.
