# Supplementary figures and images for: Effective dose to immune cells combined with platelet-to-lymphocyte ratio predicts lymphopenia and prognosis in unresectable locally advanced non-small cell lung cancer
Source: Front Immunol. 2025 Sep 24;16:1657972. doi: 10.3389/fimmu.2025.1657972 (PMC12504879; doi:10.3389/fimmu.2025.1657972)

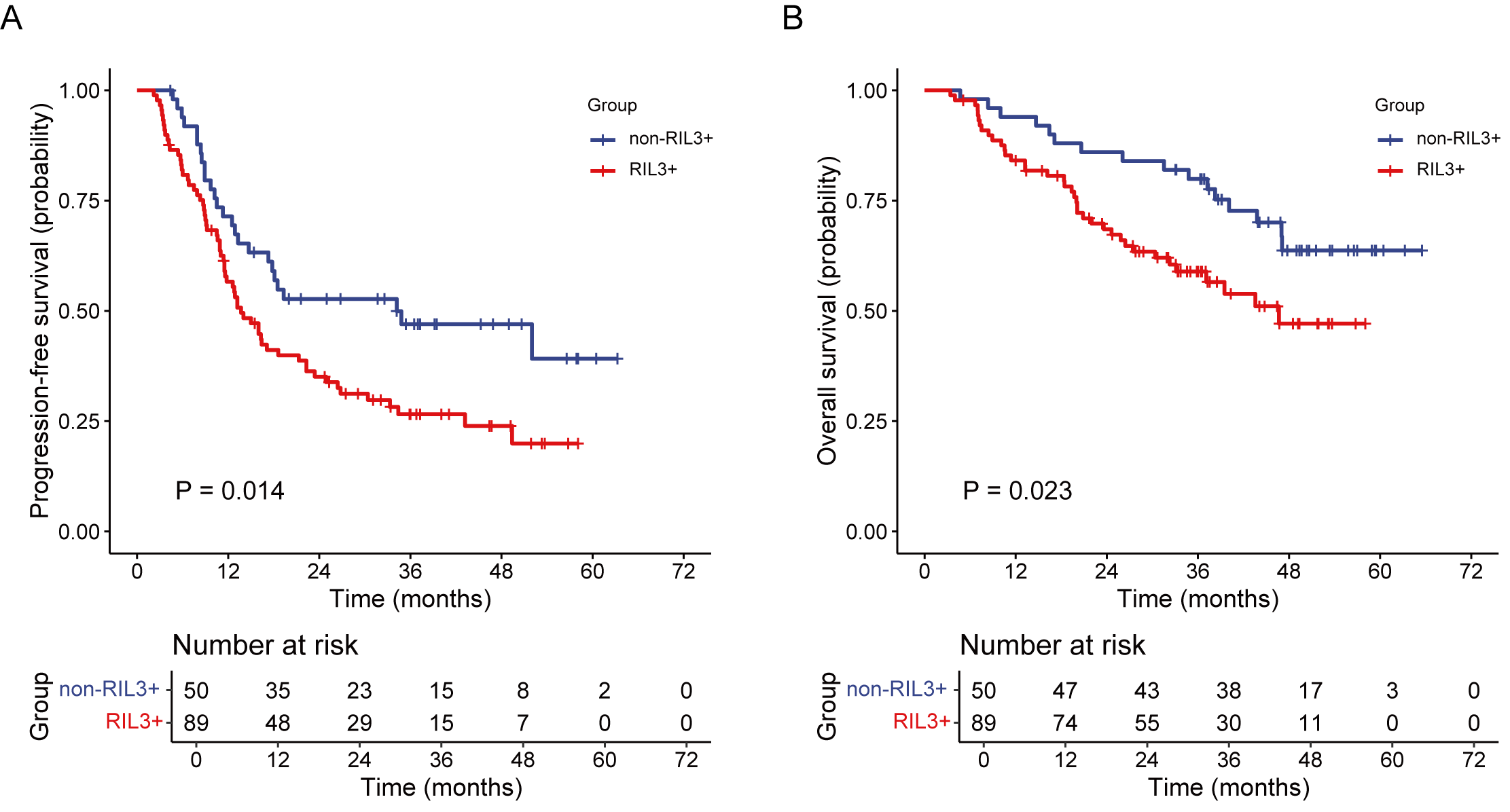

Supplement: Supplementary file 1 [file Image1.tif]

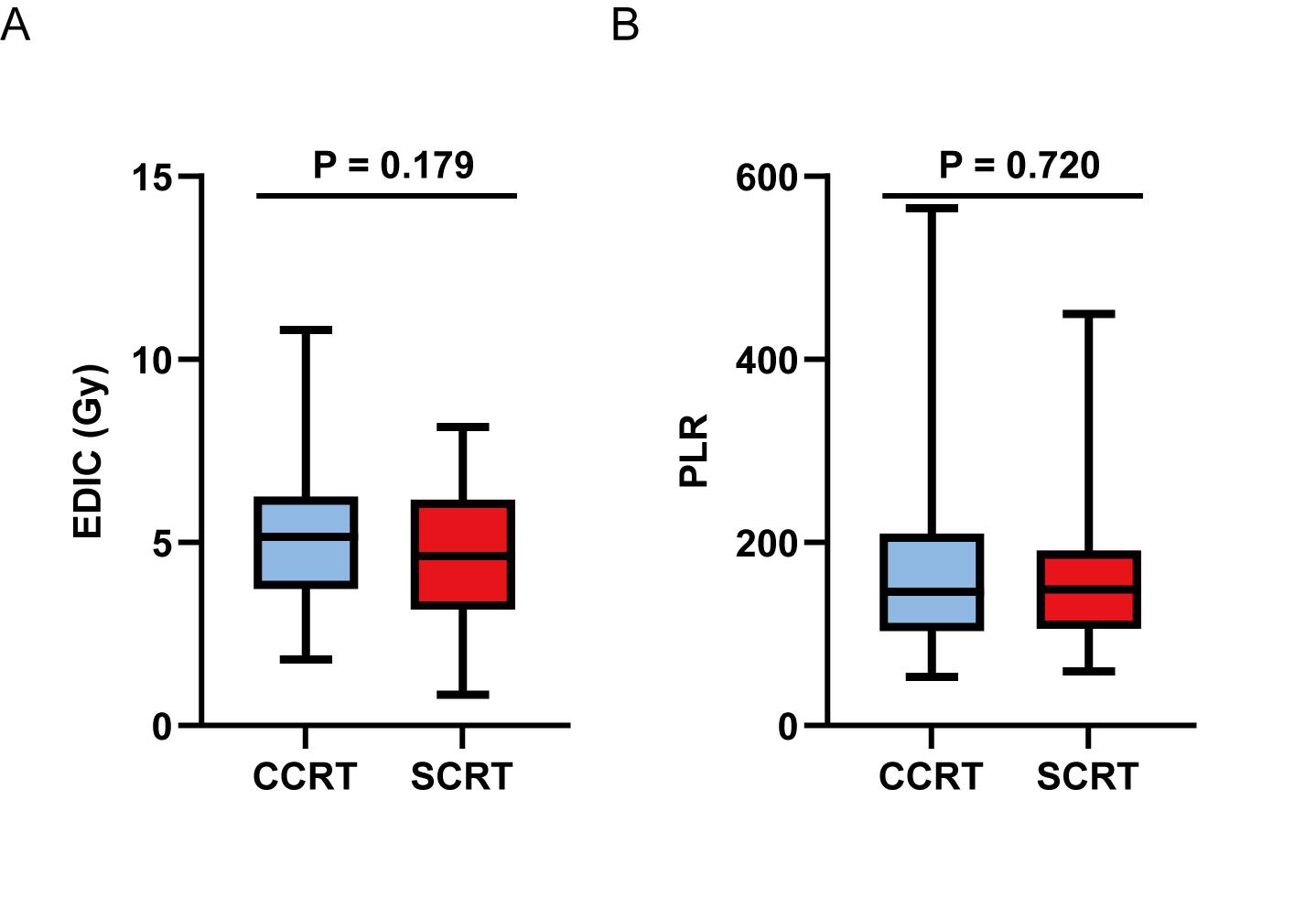

Supplement: Supplementary file 2 [file Image2.tif]

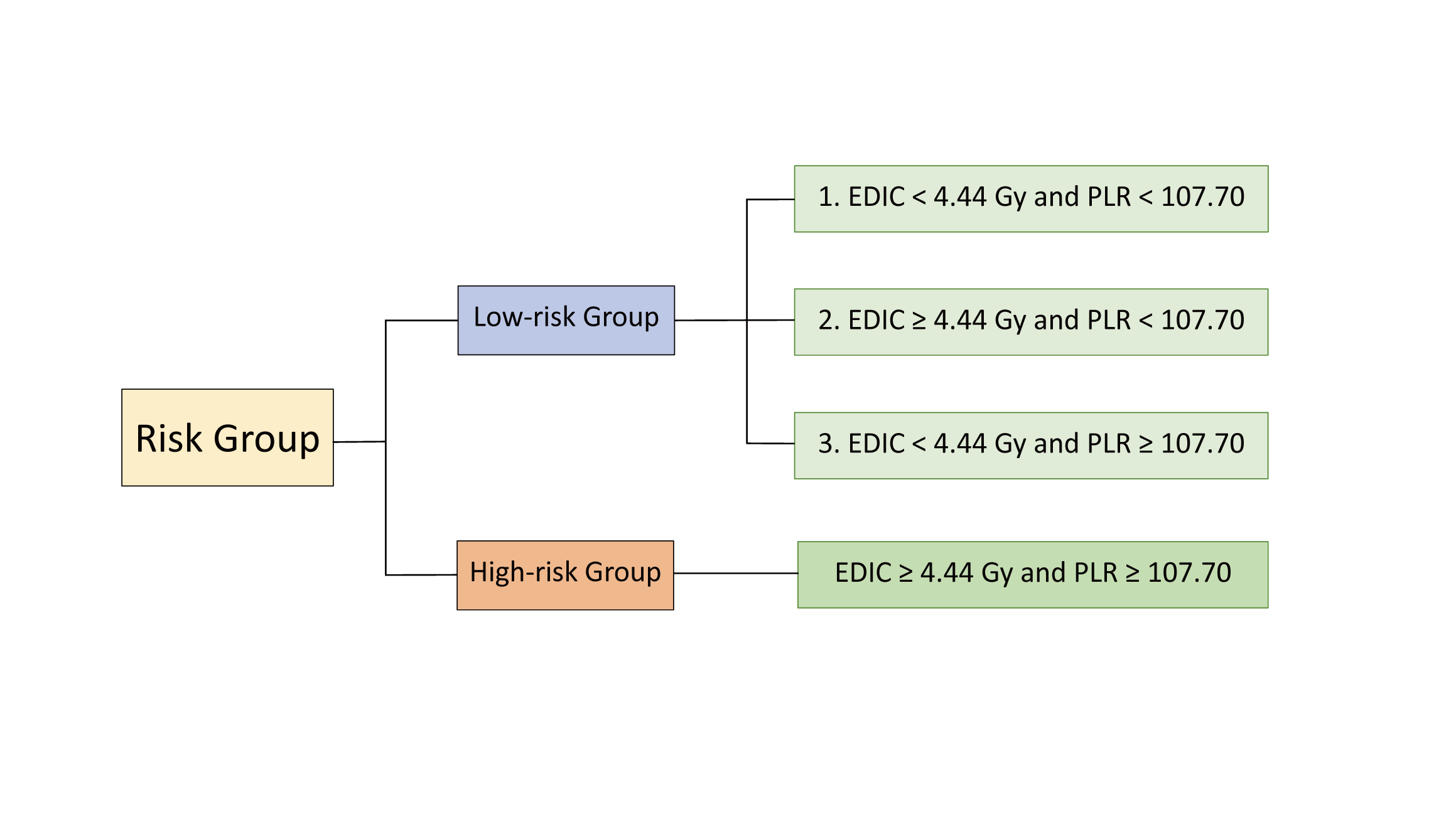

Supplement: Supplementary file 3 [file Image3.tif]

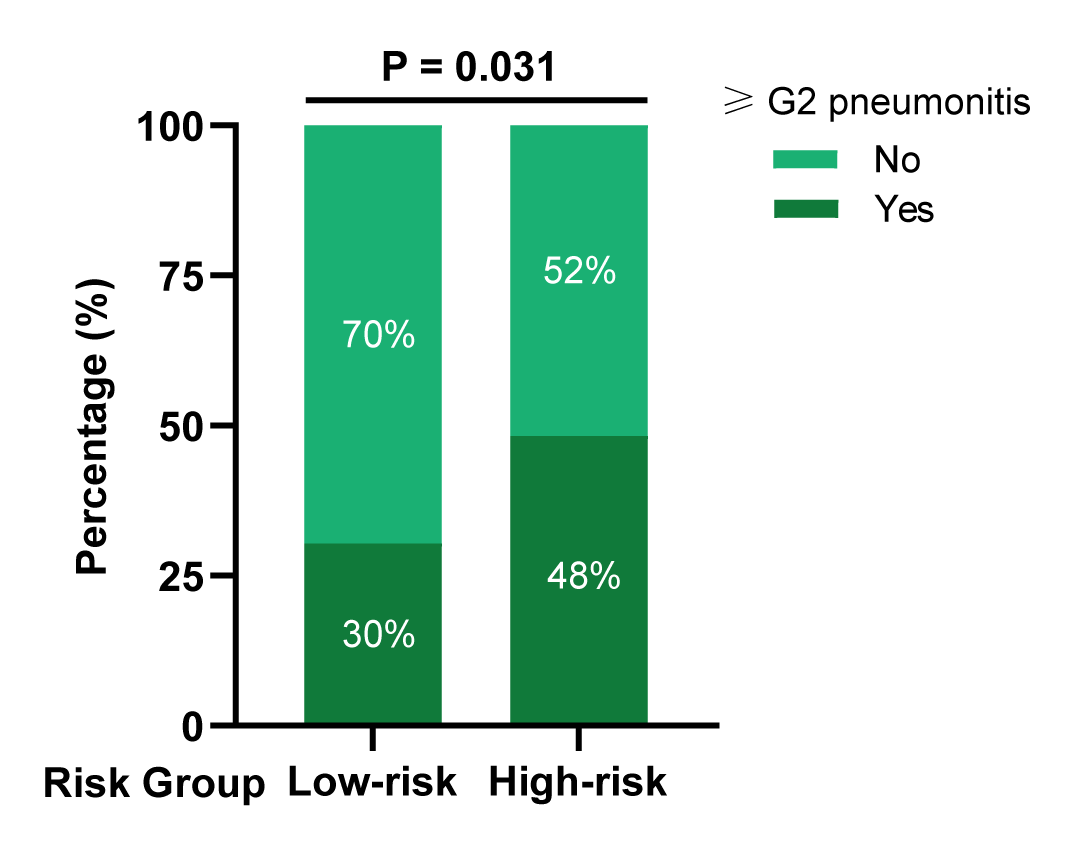

Supplement: Supplementary file 4 [file Image4.tif]
