# Supplementary material for: Effective dose to immune cells combined with platelet-to-lymphocyte ratio predicts lymphopenia and prognosis in unresectable locally advanced non-small cell lung cancer
Source: Front Immunol. 2025 Sep 24;16:1657972. doi: 10.3389/fimmu.2025.1657972 (PMC12504879; doi:10.3389/fimmu.2025.1657972)
Supplement: Supplementary file 5 [file Table1.docx]

**Supplementary Table S1.** The area under curves (AUCs) for each predictor by bootstrapping.

| **Variables** | **AUC (95%CI)** | | |
| --- | --- | --- | --- |
|  | **Apparent** | | **Bootstrapping (Optimism-corrected)** |
| **EDIC** | | 0.682 (0.589–0.682) | 0.682 (0.573–0.776) |
| **PLR** | | 0.667 (0.572–0.667) | 0.665 (0.563–0.762) |
| **EDIC+PLR** | | 0.777 (0.695–0.777) | 0.777 (0.690–0.856) |

Abbreviations: AUC, area under curve; CI, confidence interval
