# Supplementary material for: Effective dose to immune cells combined with platelet-to-lymphocyte ratio predicts lymphopenia and prognosis in unresectable locally advanced non-small cell lung cancer
Source: Front Immunol. 2025 Sep 24;16:1657972. doi: 10.3389/fimmu.2025.1657972 (PMC12504879; doi:10.3389/fimmu.2025.1657972)
Supplement: Supplementary file 6 [file Table2.docx]

**Supplementary Table S2. Patient and treatment characteristics in low-risk and high-risk groups.**

| **Characteristics** | **Low-risk Group**  **N=79 (%)** | **High-risk Group**  **N=60 (%)** | ***P value*** |
| --- | --- | --- | --- |
| **Age (y)** |  |  |  |
| < 65 | 32 (40.5) | 21 (35.0) | 0.508 |
| ≥ 65 | 47 (59.5) | 39 (65.0) |  |
| **Gender** |  |  |  |
| Male | 70 (88.6) | 47 (78.3) | 0.100 |
| Female | 9 (11.4) | 13 (21.7) |  |
| **Smoking history** |  |  |  |
| No | 34 (43.0) | 27 (45.0) | 0.817 |
| Yes | 45 (57.0) | 33 (55.0) |  |
| **ECOG** |  |  |  |
| 0 | 45 (57.0) | 35 (58.3) | 0.871 |
| 1 | 34 (43.0) | 25 (41.7) |  |
| **BMI** |  |  |  |
| < 25 | 48 (60.8) | 40 (66.7) | 0.474 |
| ≥ 25 | 31 (39.2) | 20 (33.3) |  |
| **T stage** |  |  |  |
| T1 | 11 (13.9) | 10 (16.7) |  |
| T2 | 28 (35.4) | 14 (23.3) | 0.143 |
| T3 | 15 (19.0) | 7 (11.7) |  |
| T4 | 25 (31.6) | 29 (48.3) |  |
| **N stage** |  |  |  |
| N0 | 10 (12.7) | 4 (6.7) |  |
| N1 | 13 (16.5) | 4 (6.7) | 0.126 |
| N2 | 31 (39.2) | 33 (55.0) |  |
| N3 | 25 (31.6) | 19 (31.7) |  |
| **TNM stage** |  |  |  |
| IIA-IIB | 10 (12.7) | 2 (3.3) |  |
| IIIA | 31 (39.2) | 17 (28.3) | 0.070 |
| IIIB | 30 (38.0) | 32 (53.3) |  |
| IIIC | 8 (10.1) | 9 (15.0) |  |
| **Histology** |  |  |  |
| Adenocarcinoma | 18 (22.8) | 23 (38.3) | 0.042 |
| Squamous cell carcinoma | 60 (75.9) | 34 (56.7) |  |
| Others | 1 (1.3) | 3 (5.0) |  |
| **Chemotherapy regimen** |  |  |  |
| Pemetrexed + Platinum | 18 (22.8) | 21 (35.0) | 0.262 |
| Paclitaxel + Platinum | 43 (54.4) | 26 (43.3) |  |
| Others | 18 (22.8) | 13 (21.7) |  |
| **Immunotherapy type** |  |  |  |
| Anti-PD-1 | 72 (91.1) | 48 (80.0) | 0.058 |
| Anti-PD-L1 | 7 (8.9) | 12 (20.0) |  |
| **Immunotherapy cycles** | 9 (4-15) | 8 (3-11) | 0.064 |
| **Chemotherapy cycles** | 5 (4-7) | 4 (4-6) | 0.119 |
| **PTV (cc)** | 201.20 (119.50-310.00) | 272.05 (208.80-384.50) | 0.109 |
| **Radiation dose (Gy)** | 60.00 (58.00-60.00) | 60.00 (58.75-60.00) | 0.862 |
| **EDIC** | 3.77 (3.08-5.23) | 5.88 (5.10-6.72) | ＜0.001 |
| **Baseline blood markers** |  |  |  |
| NLR | 2.45 (1.41-3.67) | 2.55 (1.73-3.65) | 0.904 |
| dNLR | 1.74 (1.13-2.70) | 1.78 (1.29-2.69) | 0.737 |
| LMR | 3.79 (2.65-5.48) | 3.30 (2.43-5.63) | 0.210 |
| PLR | 127.20 (85.69-185.56) | 154.17 (131.78-229.03) | ＜0.001 |
| SII | 546.39 (258.88-844.90) | 557.48 (388.23-831.37) | 0.143 |

Abbreviations: ECOG, Eastern Cooperative Oncology Group; BMI, body mass index; PTV, planning targrt volume; EDIC, effective dose to immune cells; ALC, absolute lymphocyte count; NLR, neutrophil-to-lymphocyte ratio; dNLR, derived neutrophil-to-lymphocyte ratio; LMR, lymphocyte-to-monocyte ratio; PLR, platelet-to-lymphocyte ratio; SII, systemic immune-inflammation index

#Values are number (percentage) or median (interquartile range).
