# Supplementary material for: Effective dose to immune cells combined with platelet-to-lymphocyte ratio predicts lymphopenia and prognosis in unresectable locally advanced non-small cell lung cancer
Source: Front Immunol. 2025 Sep 24;16:1657972. doi: 10.3389/fimmu.2025.1657972 (PMC12504879; doi:10.3389/fimmu.2025.1657972)
Supplement: Supplementary file 7 [file Table3.docx]

Supplementary Table S3. IrAEs spectrum in low-risk and high-risk groups.

| IrAEs | Low-risk Group | | High-risk Group |
| --- | --- | --- | --- |
| All irAEs | 34 | 30 | |
| Thyroid dysfunction | 15 | 10 | |
| CIP | 5 | 9 | |
| Hepatic toxicity | 7 | 5 | |
| Hematologic toxicity | 2 | 3 | |
| Skin toxicity | 4 | 2 | |
| Diarrhea/colitis | - | 1 | |
| Hydroncus | 1 | - | |

Abbreviations: CIP, checkpoint inhibitor-associated pneumonitis
