# Supplementary material for: Effective dose to immune cells combined with platelet-to-lymphocyte ratio predicts lymphopenia and prognosis in unresectable locally advanced non-small cell lung cancer
Source: Front Immunol. 2025 Sep 24;16:1657972. doi: 10.3389/fimmu.2025.1657972 (PMC12504879; doi:10.3389/fimmu.2025.1657972)
Supplement: Supplementary file 8 [file Table4.docx]

**Supplementary Table S4. Sensitivity analysis of PLR cut-offs.**

|  | **OR (95%CI)** | | ***P* value** |
| --- | --- | --- | --- |
| **PLR** |  |  | |
| **＜107.7** | 1.00 (Reference) |  | |
| **≥107.7** | 5.40 (2.40-12.11) | <0.001 | |
| **PLR** |  |  | |
| **＜144** | 1.00 (Reference) |  | |
| **≥144** | 2.31 (1.14-4.70) | 0.020 | |
| **PLR** |  |  | |
| **＜169** | 1.00 (Reference) |  | |
| **≥169** | 2.05 (0.94-4.46) | 0.069 | |

Abbreviations: PLR, platelet-to-lymphocyte ratio; OR: odds ratio; CI: confidence interval
